# Deciphering hematopoietic stem cell development: key signaling pathways and mechanisms

**DOI:** 10.3389/fcell.2024.1510198

**Published:** 2024-12-09

**Authors:** Saori Morino-Koga, Tomomasa Yokomizo

**Affiliations:** ^1^ Department of Cell Differentiation, Institute of Molecular Embryology and Genetics, Kumamoto University, Kumamoto, Japan; ^2^ Microscopic and Developmental Anatomy, Tokyo Women’s Medical University, Tokyo, Japan

**Keywords:** intra-aortic hematopoietic cluster (IAHC), hematopoietic stem cell (HSC), erythro-myeloid progenitor (EMP), endothelial-to-hematopoietic transition (EHT), hematopoietic stem cell precursor (pre-HSC), aorta-gonad-mesonephros (AGM)

## Abstract

Most blood cells derive from hematopoietic stem cells (HSCs), originating from endothelial cells. The induction of HSCs from endothelial cells occurs during mid-gestation, and research has revealed multiple steps in this induction process. Hemogenic endothelial cells emerge within the endothelium, transition to hematopoietic cells (pre-HSCs), and subsequently mature into functional HSCs. Reports indicate transcription factors and external signals are involved in these processes. In this review, we discuss the timing and role of these transcription factors and summarize the external signals that have demonstrated efficacy in an *in vitro* culture. A precise understanding of the signals at each step is expected to advance the development of methods for inducing HSCs from pluripotent stem cells.

## Introduction

Various types of blood cells, such as lymphoid and myeloid cells, are produced from hematopoietic stem cells (HSCs) in the bone marrow, which is not the site of HSC development. During mouse embryogenesis, HSC development begins with endothelial cell differentiation. Some endothelial cells undergo an endothelial-to-hematopoietic transition (EHT), giving rise to HSC-precursors (pre-HSCs) (Taoudi et al., 2008; Zhou et al., 2016). The pre-HSCs then migrate to the fetal liver and mature into HSCs, which ultimately move to the bone marrow and maintain lifelong hematopoiesis (Dzierzak and Speck, 2008; Orkin and Zon, 2008).

HSCs arise from endothelial cells in the aorta-gonad-mesonephros (AGM) region (Muller et al., 1994; Cumano et al., 1996; Medvinsky and Dzierzak, 1996; de Bruijn et al., 2002; Zovein et al., 2008). Although the HSC developmental process appears simple, there are branching points at each differentiation stage that determine the cell fate. Most endothelial cells engage in the formation of blood vessels, but a small proportion of these cells differentiate into hemogenic endothelial cells. These hemogenic endothelial cells undergo an EHT and differentiate into pre-HSCs as well as hematopoietic progenitor cell precursors (pre-HPCs), which enter into processes that lead to the formation of intra-aortic hematopoietic clusters (IAHCs) in the dorsal aorta of the AGM region between the embryonic day (E) 9.5 and E11.5 (Zovein et al., 2008; Chen et al., 2009; Boisset et al., 2010; Yokomizo and Dzierzak, 2010; Boisset et al., 2015). Here, we focus on the transcription factors and their target genes, and discuss hemogenic endothelium development, EHT, and IAHC formation. We also summarize the signaling molecules that are expressed during HSC development and the timing of their expression, focusing primarily on findings obtained from *in vitro* culture studies.

## Developmental pathway of HSCs

### Development of hemogenic endothelium

The first HSCs were observed in the AGM region during midgestation (Muller et al., 1994; Medvinsky and Dzierzak, 1996), and were shown to originate from IAHCs derived from endothelial cells in the innermost layer of the AGM region (Jaffredo et al., 1998; Zovein et al., 2008; Boisset et al., 2010). Whereas most endothelial cells eventually contribute to blood vessel formation, some differentiate into hemogenic endothelium, which undergoes an EHT to generate IAHCs. Amongst several transcription factors, RUNX1 has been shown to be a key factor during this sequence. RUNX1 is important for hematopoietic development and is lethal in knockout mice around E12.5 due to extensive hemorrhages (Okuda et al., 1996). North et al. showed the generation of HSCs from *Runx1*-expressing cells (North et al., 2002), with RUNX1 contributing to HSC production via an enhancer regulated by GATA/ETS/SCL (Nottingham et al., 2007). The activation of this enhancer was initiated in the endothelial cell layer, suggesting that hemogenic endothelium development likely occurs in the endothelial cells (Swiers et al., 2013). Indeed, when *Runx1* is deleted in cells expressing the endothelial cell markers *Cdh5* or *Tie2*, HSCs are not generated (Li et al., 2006; Chen et al., 2009), indicating that endothelial cells are the origin of HSCs and that RUNX1 contributes to their developmental process.

RUNX1 functions as a transcription factor by forming a dimer with CBFB; thus, *Cbfb*-deficient mice exhibit the same phenotype of definitive hematopoietic deficiency as *Runx1*-deficient mice. Chen et al. attempted to rescue this hematopoietic defect in *Cbfb*-deficient mice by introducing transgenes but found that whereas the introduction of *Tie2-Cbfb* induced erythro-myeloid progenitors (EMPs), only the introduction of *Ly6a-Cbfb* induced HSCs (Chen et al., 2011). These findings strongly suggest that hemogenic endothelial cells producing EMPs and HSCs are distinct.

In the yolk sac, EOMES transcription factor is expressed earlier than RUNX1, and regulates hematopoietic cell production through RUNX1 (Harland et al., 2021). Additionally, *Meis1* is reported to be expressed before RUNX1 in the AGM region, and is important in regulating pre-hemogenic endothelium development (Coulombe et al., 2023). *Meis1*-knockout mice show reduced hematopoietic cells, including HSC production, and are embryonic lethal by E14.5 due to defective hematopoiesis and angiogenesis (Hisa et al., 2004; Azcoitia et al., 2005). Thus, studies suggest that fate determination from endothelial cells to hemogenic endothelial cells is initiated prior to the expression of RUNX1, and further elucidation of the mechanism underlying hemogenic endothelium development is necessary to expound upon these findings.

The search for hemogenic endothelium markers working alongside RUNX1 is ongoing (Fadlullah et al., 2022). Several groups are focusing on cell-surface markers to isolate select cells for experimentation. CD44 is expressed in endothelial cells, including hemogenic endothelial cells, and is also maintained in IAHCs formed by EHT from hemogenic endothelium (Oatley et al., 2020). Recently, it was reported that CD32 is characteristically expressed in hemogenic endothelial cells in human embryonic and human iPS-derived endothelial cells (Scarfo et al., 2024). Many CD32^+^ endothelial cells differentiate into hematopoietic cells, which enriches the hemogenic endothelium. However, it remains unclear whether CD32^+^ hemogenic endothelial cells can differentiate into HSCs, because an *in vitro* system that demonstrates the differentiation of human hemogenic endothelial cells into HSCs has not been established. Other works indicate the existence of different types of hemogenic endothelial cells (Chen et al., 2011; Dignum et al., 2021; Kobayashi et al., 2023), and thus we expect to see future work describing HSC-specific hemogenic endothelial cells.

### Molecular mechanism of EHT

EHT is based on two events: the loss of endothelial cell characteristics and the acquisition of hematopoietic cell characteristics. As IAHCs were not formed from hemogenic endothelium in *Runx1*-deficient mouse embryos (North et al., 1999; Yokomizo et al., 2001), many studies have since sought to investigate the role of RUNX1 in EHT. In an *in vitro* differentiation system using ES cells, one group showed that the loss of *Gfi1* and *Gfi1b*—the target genes of RUNX1—results in the persistence of endothelial cell morphology instead of the spherical shape characteristic of hematopoietic cells (Lancrin et al., 2012). The same group has also shown that the loss of *Gfi1* and *Gfi1b* in the AGM region prevents EHT and the formation of IAHCs, and thus a failure to produce HSCs (Thambyrajah et al., 2016). In their report, the authors showed that *Gfi1* is specifically expressed in hemogenic endothelium from E10.5 and that its expression gradually decreases. However, contrastingly, the expression of *Gfi1b* increases, and this is consistent with the expression pattern of hematopoietic cell markers, such as KIT and CD41. The authors also show that a complex comprising LSD1 generates epigenetic changes that contribute to the loss of endothelial cell characteristics by GFI1 and GFI1B. Yet, despite these findings, inducing *Gfi1* and *Gfi1b* expression in *Runx1* KO cells to produce hematopoietic-like spherical cells leads to low colony-forming capacity. Collectively, these results suggest that molecules other than GFI1 and GFI1B might be involved in the acquisition of hematopoietic cell characteristics (Lancrin et al., 2012).

In parallel with the loss of endothelial cell characteristics by GFI1 and GFI1B, several hematopoietic-related genes become activated. ETS transcription factor *Spi1* is one of the target genes activated by RUNX1 (Okada et al., 1998; Huang et al., 2008; Hoogenkamp et al., 2009). In *Spi1*-deficient mice, there is a reduction in the proportion of pre-HSCs (CD31^+^KIT^+^CD45^+^ cells) in the AGM region as well as HSCs in the fetal liver (Kim et al., 2004; Zhang et al., 2024). The requirement of SPI1 for the acquisition of hematopoietic competence is conserved across species. Single-cell RNA-sequencing (scRNA-seq) data analyses of the human fetal AGM region or of endothelial cells and hematopoietic cells derived from the human iPS cells show that the decrease in endothelial cell marker genes, *Cdh5* and *Sox17*, is accompanied by an increase in *Runx1*, *Spi1*, and *Gata2* (Qu et al., 2024). *Spi1* regulates the heterogeneity of hematopoietic cell differentiation pathways, and the *Spi1* target genes, *Lyl1* and *Klf1*, determine the direction of hematopoietic cell differentiation (Qu et al., 2024).

GATA2 is another transcription factor that regulates EHT. Deletion of the transcriptional regulatory region of *Gata2* can inhibit EHT and the formation of IAHCs (Gao et al., 2013). Interestingly, the authors show that repression of *Gata2* transcription activity can suppress the expression of other transcription factors, such as *Runx1* and *Tal1*. Previous reports showed that deletion of *Tal1* causes embryonic lethality due to hematopoietic failure and TAL1 contributes to erythroid differentiation (Shivdasani et al., 1995; Mikkola et al., 2003). Thus, it is known that there are multiple transcription factors involved in EHT, and they cause EHT in a coordinated manner. This intricate network of transcription factors has been shown to continuously play a crucial role throughout the developmental process of HSCs (Wilson et al., 2010). On the other hand, studies using zebrafish and mice have also reported that GATA2 causes HSC development independently of RUNX1 (Bresciani et al., 2021). *Gata2*-knockout mice have been reported to die of hematopoietic failure at around E10.5 (Tsai et al., 1994), and *Gata2* heterozygous knockout mice also show abnormalities in HSC generation and function (Ling et al., 2004). More interestingly, *Gata2* loss specifically in *Cdh5*-expressing endothelial cells inhibits HSC generation, and *Gata2* loss in *Vav*-expressing hematopoietic cells after EHT results in an inability to maintain HSCs. These reports suggest that GATA2 plays a dual role in the regulation of HSCs from their generation to their maintenance (de Pater et al., 2013). On the other hand, the deletion of *Runx1* specifically in *Vav*-expressing hematopoietic cells does not reduce HSCs (Chen et al., 2009), suggesting that RUNX1 is important for differentiation progression up to EHT before E11.5.

Regarding EHT process, many scRNA-seq analyses have been conducted using pre- and post-EHT cells to explore its mechanisms and specific markers (Zhou et al., 2016; Baron et al., 2018; Hou et al., 2020; Vink et al., 2020; Zhu et al., 2020; Calvanese et al., 2022; Fadlullah et al., 2022; Hadland et al., 2022; Yokomizo et al., 2022; Menegatti et al., 2023). These analyses have confirmed the dynamic expression patterns of transcription factors such as *Runx1* and *Gfi1*, which have previously been implicated in EHT. Furthermore, similarities between mice and humans have been observed in the expression of HSC-related transcription factors (*Hlf* and *Mecom*) (Zhou et al., 2016; Calvanese et al., 2022; Yokomizo et al., 2022). However, no mention has been made regarding the potential involvement of novel transcription factors in EHT. On the other hand, these studies have been highly effective in identifying new surface markers. Hadland et al. identified VE-cadherin^+^CD61^+^EPCR^+^ cells as a precursor to functional HSCs (Hadland et al., 2022), Vink et al. reported that the earliest functional HSCs are marked by CD27 (Vink et al., 2020), and Menegatti et al. identified CD82 as a novel surface marker specific to EHT (Menegatti et al., 2023).

### Formation of IAHCs and their heterogeneity

EHT causes the formation of specific cell clusters in arteries called IAHCs (Boisset et al., 2010; Yokomizo and Dzierzak, 2010). The cells constituting these IAHCs express the transcription factor *Hlf*, the expression of which is maintained after the cells differentiate into HSCs (Yokomizo et al., 2019). *Hlf* expression is regulated by the transcription factor *Evi1*, as evidenced by the reduction in *Hlf*-positive cells in *Evi1*-deficient mice. Thus, *Evi1* activation and the subsequent expression of *Hlf* in cells within IAHCs may be a pathway for HSC differentiation. Interestingly, *Evi1* expression is heterogeneous within IAHCs and cells with high *Evi1* expression can differentiate into HSCs (Yokomizo et al., 2022). Therefore, it is possible that cells with high *Evi1* expression within IAHCs are pre-HSCs. Future studies are expected to reveal the differentiation pathway of pre-HSCs to HSCs using a combination of transcription factors and cell-surface antigens as markers.

**FIGURE 1:**
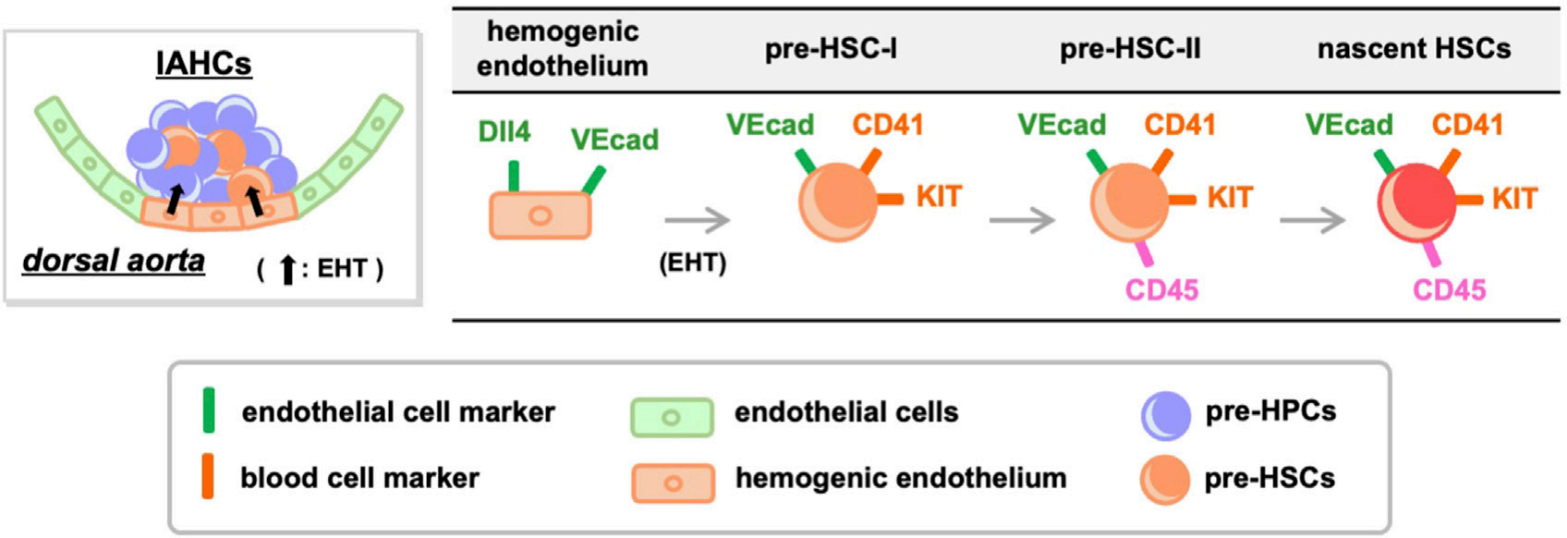
Transition of cell-surface antigens during HSC development. Hemogenic endothelium expressing endothelial cell markers undergoes EHT and differentiates into pre-HSC-I and pre-HPCs. During this process, some endothelial cell markers are retained while the expression of blood cell markers increases. Pre-HSC-I then differentiates into pre-HSC-II, which express CD45, and ultimately mature into HSCs. VEcad, VE-cadherin.

HSC development has been defined using cell-surface antigens (Figure 1). Hemogenic endothelial cells transform into hematopoietic cells by EHT, and demonstrate a significant change in cell-surface antigen expression. Hemogenic endothelium before EHT expresses the endothelial cell marker VE-cadherin and the arterial endothelial cell marker DLL4, while the blood cell markers KIT, CD41, and CD45 are not yet expressed (Hadland et al., 2015; Hadland et al., 2017; Morino-Koga et al., 2024). When EHT occurs and IAHCs are formed, DLL4 expression is downregulated (Porcheri et al., 2020) and this downregulation is inversely correlated with the increased expression of KIT and CD41. On the contrary, VE-cadherin expression is maintained after EHT (Rybtsov et al., 2011; Rybtsov et al., 2014; Porcheri et al., 2020; Morino-Koga et al., 2024). Cells that eventually become CD45^+^ and migrate to the liver will acquire the ability to become HSCs (Rybtsov et al., 2011; Rybtsov et al., 2014; Morino-Koga et al., 2024). After liver migration, VE-cadherin expression gradually decreases and HSCs expressing so-called HSC markers are detected (Morrison et al., 1995; Kim et al., 2005; Taoudi et al., 2005; Papathanasiou et al., 2009).

Of note, IAHCs are not a cell mass containing only pre-HSCs but also contain heterogeneous cell populations, most of which are pre-HPCs that do not differentiate into HSCs (Baron et al., 2018; Yokomizo et al., 2022). Consequently, it is difficult to distinguish pre-HSCs from pre-HPCs using the current definition of pre-HSCs through cell-surface antigens. The search for specific marker molecules for pre-HSCs is an upcoming challenge.

## HSC development and signaling molecules

Thus far, HSC development via the intricate interactions of nuclear transcription factors has been summarized. The next question is, how do extracellular signals control HSC development? Unlike transcription factors, it is challenging to clarify the roles of signals because knockout mice do not exhibit clear phenotypes. A common approach is to add signaling molecules to culture systems to observe their effects. Specifically, tissues or cell populations that are precursors to HSCs are isolated from mouse embryos and transferred to culture systems where different signaling molecules are introduced to assess whether transplantable HSCs can be induced. This method helps to infer the signaling pathways functioning *in vivo*.

### SCF

After studies showed evidence for HSCs within the AGM region, many groups sought to differentiate AGM-derived cells into HSCs *in vitro*. Medvinsky’s group developed an *ex-vivo* culture system to demonstrate the existence of pre-HSCs. They co-cultured pre-HSC candidate cells (VE-cadherin^+^CD45^+^ cells) with AGM stromal elements (including endothelial cells) in the presence of stem cell factor (SCF), Fms-related tyrosine kinase 3 ligand (Flt3l), and interleukin-3 (IL-3), and showed induction of transplantable HSCs (Taoudi et al., 2008). These AGM stromal elements could be replaced by simply using the OP9 stromal cell line (Rybtsov et al., 2011), with HSCs induced from E9.5 pre-HSC-I (VE-cadherin^+^CD45^−^CD41^lo^ cells) (Rybtsov et al., 2014). This report also examined the necessity for signaling molecules, and showed that E9.5 pre-HSC-I differentiated into HSCs after supplementing SCF with OP9 stromal cells and serum, and this occurred even in the absence of other signaling molecules. Others have shown production of HSCs from E9.5 hemogenic endothelium under serum-free culture conditions following the addition of four signaling factors—SCF, Flt3l, IL-3, and thrombopoietin (TPO)—as well as AGM-derived endothelial feeder cells overexpressing Akt (Hadland et al., 2015). More recently, we showed that E11.5 pre-HSC-I differentiated into HSCs in the presence of SCF and TPO, even under serum-free and feeder-free conditions (Morino-Koga et al., 2024). These results indicate that SCF consistently contributes to HSC development from E9.5. KIT, an SCF receptor, is persistently expressed from E9.5 pre-HSC-I to E12.5 HSCs (Hadland et al., 2015; Morino-Koga et al., 2024), suggesting that SCF is continuously required for HSC development.

*Scf* deficiency is known to cause perinatal lethality due to severe anemia (Ding et al., 2012), and loss of *Scf* activity can impair HSC generation and function (Azzoni et al., 2018). Therefore, SCF might be important for HSC development and hematopoietic function. However, does SCF reside in the AGM region? Through scRNA-seq of periaortic tissues and livers from E10.5 and E11.5 mouse embryos, we recently revealed that *Scf* is expressed in various tissues, including endothelial cells, stromal cells, genital ridge progenitor cells, nephric duct, and hepatoblasts (Morino-Koga et al., 2024). These results are consistent with the earlier histological findings showing *Scf* expression to be strongly observed in the ventral wall of the dorsal aorta (Souilhol et al., 2016a). In that report, *Scf* expression was also detected in surrounding cells, such as stromal cells and genital ridge cells, supporting that *Scf* is present in the microenvironment where IAHC formation occurs. Among these, *Scf* produced by endothelial cells is important for HSC development (Azzoni et al., 2018).

### Flt3l

Flt3l was identified as a ligand for FLT3 (Lyman et al., 1993) and, since *Flt3*-deficient mice exhibit hematopoietic abnormalities (Mackarehtschian et al., 1995), it is thought to be involved in lineage commitment. Additionally, FLT3 is often used as a marker for hematopoietic progenitors immediately after differentiation from HSCs (Adolfsson et al., 2005; Forsberg et al., 2006). Tracing experiments using *Flt3-Cre* BAC transgenic mice have shown that nearly all hematopoietic lineages, except HSCs, are marked (Boyer et al., 2011; Buza-Vidas et al., 2011). Interestingly, a subset of HSCs in the embryonic stage is marked by *Flt3-Cre*, but these FLT3^+^ cell-derived HSCs disappear after birth (Beaudin et al., 2016). These results suggest that Flt3l/FLT3 signaling is not essential for the development and maintenance of the types of HSCs that are preserved into adulthood. On the other hand, in HSC induction culture systems, Flt3l is added along with SCF and IL-3 and is used for the induction of HSCs from pre-HSCs after E9.5 (Rybtsov et al., 2011; Rybtsov et al., 2014). In an aggregation culture system developed later using OP9 stromal cells, the authors suggested that Flt3l was not needed (Rybtsov et al., 2014) and suggested that Flt3l may not be essential for the *in vitro* development (maturation) of HSCs. Indeed, it is possible that the same effect afforded by Flt3l is compensated by other pathways.

### IL-3

Early findings by Robin et al. highlighted IL-3 as an inducer of transplantable HSCs in an *in vitro* culture system using E11.5 AGM region isolated from *Runx1* ^+/−^ mice (Robin et al., 2006). Since then, IL-3 is touted to play a role in HSC development through RUNX1. In the same report, the authors also showed that lL-3 was expressed in the cells of the AGM region, with expression of the IL-3 receptor noted on some hematopoietic cells in that region. Later work showed IL-3 as an effective inducer of HSC differentiation of E11.5 pre-HSCs in a co-aggregation culture with OP9 stromal cells (Rybtsov et al., 2014). Collectively, these findings suggest a contribution by IL-3 in the differentiation of pre-HSCs into HSCs after E11.5.

### Inflammatory signaling

Inflammatory signaling has been reported to be involved in the development and maintenance of HSCs during embryogenesis (Espin-Palazon et al., 2014; Sawamiphak et al., 2014; He et al., 2015; Mariani et al., 2019; Frame et al., 2020; Zhang et al., 2024). Both *Interferon-γ (Ifn-γ)*- and *Ifn-γ* receptor-deficient mouse embryos show a reduced proportion of functional HSCs (Li et al., 2014). However, bone marrow-derived HSCs in *Ifn-γ*-deficient mice are normal (Baldridge et al., 2010), suggesting that the HSCs produced are functional. It is surmised that the addition of IFN-γ may be effective in inducing functional HSCs *in vitro*. One group showed that the addition of interferon-α (IFN-α) to AGM-derived cells using an *in vitro* culture system could induce HSCs with high engraftment capacity in the bone marrow (Kim et al., 2016). In that report, *Ifn-α* was minimally expressed in the E11.5 AGM region but upregulated in the E13.5 fetal liver, suggesting IFN-α involvement in the maturation of HSCs in the fetal liver.

### Notch signaling

Recently, we reported that E11.5 pre-HSC-I could induce HSCs by adding only SCF and TPO even in the absence of serum and feeder cells (Morino-Koga et al., 2024). However, we also showed that E10.5 pre-HSC-I and E10.5 hemogenic endothelium could not differentiate into HSCs with SCF and TPO, requiring co-culture with the endothelial cell line. These results are consistent with previous results obtained using Akt-overexpressing embryonic endothelial cell lines (Hadland et al., 2015; Hadland et al., 2017), suggesting that co-culture with endothelial cells might be necessary to induce HSCs from E10.5 hemogenic endothelium in an *in vitro* culture system. Furthermore, HSCs have been successfully produced from E9.5-E10.5 CD45^−^VE-cadherin^+^CD41^-^Dll4^+^ cells, which are defined as arterial endothelial cells, by co-culturing with endothelial feeder cells (Hadland et al., 2017; Morino-Koga et al., 2024). Thus, it might support the process of generating HSCs from arterial endothelial cells via EHT.

What kind of environment do endothelial cells provide for hemogenic endothelium? One of the most promising candidates is Notch signaling (Clements et al., 2011; Kanz et al., 2016; Rho et al., 2019; Thambyrajah and Bigas, 2022). Notch signaling is also important for angiogenesis, and thus it is not easy to distinguish whether hematopoietic cells are directly affected or if their absence is due to a defect in angiogenesis. Studies using ES cells or endothelial cells derived from mice lacking *Notch1* have reported that Notch1 is required for endothelial cells to undergo EHT and differentiate into HSCs (Kumano et al., 2003; Hadland et al., 2004). These results suggest that hemogenic endothelium and/or pre-HSCs might express Notch1, since cells lacking *Notch1* cannot differentiate into HSCs. HSCs can be induced from E10.5 CD45^−^VE-cadherin^+^ cells with activated Notch signaling by aggregate culture with OP9 stromal cells and suppressed by either adding the Notch inhibitor DAPT to the AGM tissue culture at E10.5, or by treating a reaggregated culture of E10.5 AGM-derived cells with a Notch1 inhibitor antibody (Souilhol et al., 2016b). Collectively, these findings suggest that Notch1-expressing cells differentiate into HSCs. Let us return to the main question: Does the endothelial cell line express a ligand acting on the Notch1 receptor? Whole-embryo immunohistochemical staining of mice at E9.5 and E10.5 shows that endothelial cells in the P-Sp/AGM region express the Notch ligands (DLL4, JAG1, and JAG2) and receptors (Notch1 and Notch4) (Robert-Moreno et al., 2005). Notch1 is expressed in the ventral side of the dorsal aorta where IAHCs form, whereas Notch4 is uniformly expressed throughout the vessel. Therefore, these Notch1-expressing cells are expected to undergo EHT and differentiate into HSCs. On the other hand, all three Notch ligands are expressed throughout the endothelial cells. Compared with primary endothelial cells, the authors highlighted an upregulation in the expression of DLL1, DLL4, JAG1, and JAG2 in Akt-overexpressing endothelial cells (Hadland et al., 2015). In addition, endothelial cell lines that contributed to HSC development showed expression of *Dll1*, *Dll4*, and *Jag1*, but not *Jag2*; albeit the expression intensity varied (Morino-Koga et al., 2024). Thus, the endothelial cell line may not only serve as a scaffold but may also contribute to the HSC differentiation via the Notch ligands, DLL4 and JAG1.

**FIGURE 2:**
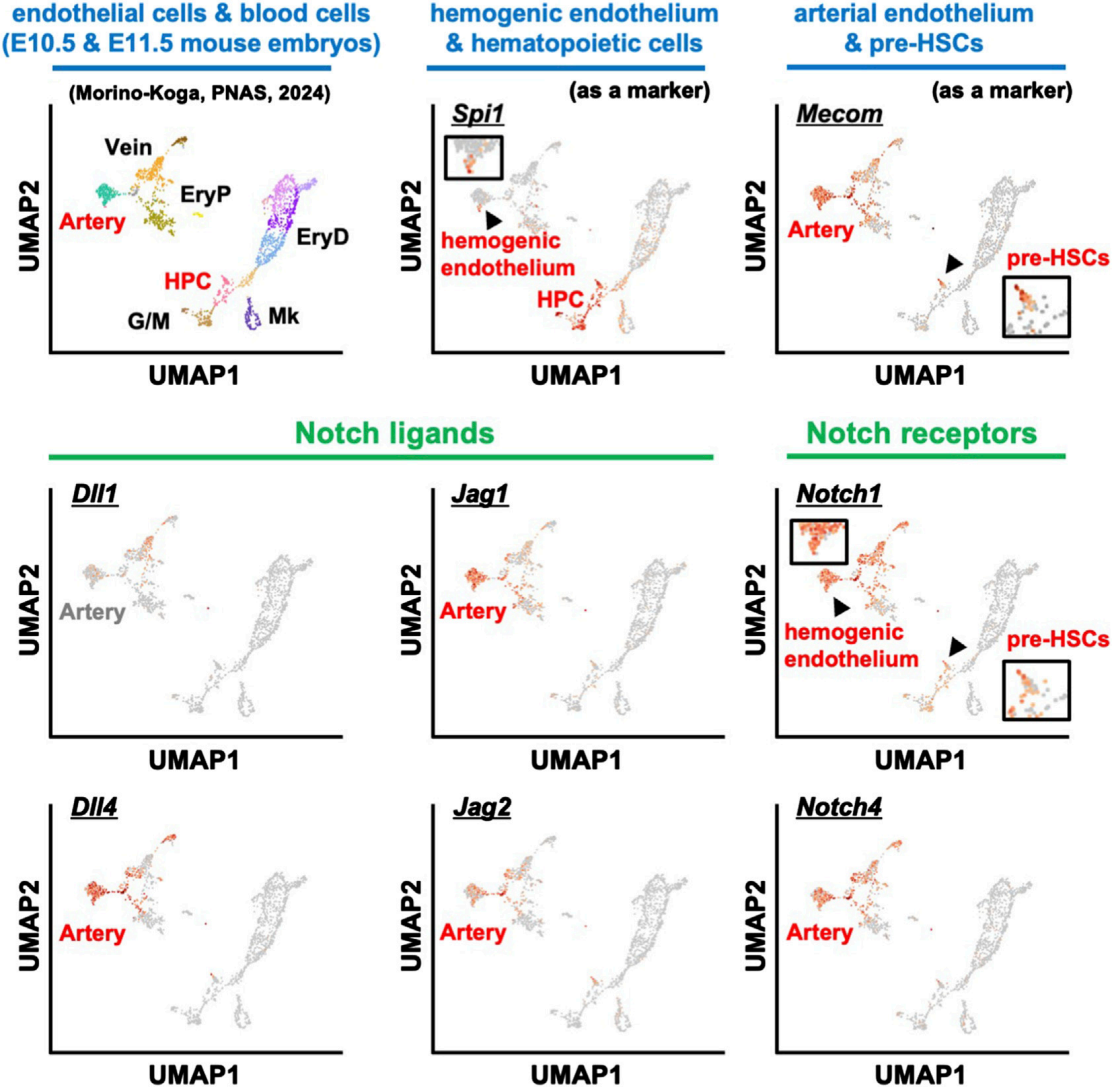
scRNA-seq analysis of blood and endothelial cells from E10.5 and E11.5 mouse embryos. The expression patterns of various marker genes: hemogenic endothelium and HPC (Spi1), endothelial cells and pre-HSCs (Mecom), Notch ligands (Dll1, Dll4, Jag1, Jag2), and Notch receptors (Notch1 and Notch4). HPC, hematopoietic progenitor cells; EryP, primitive erythrocytes; EryD, definitive erythrocytes; G/M, granulocytes and monocytes; Mk, megakaryocytes.

For Notch signaling, we analyzed the scRNA-seq dataset using endothelial cells and hematopoietic cells from E10.5-E11.5 mouse embryos (Figure 2) (Yokomizo et al., 2022; Morino-Koga et al., 2024). Consistent with previous histological findings (Robert-Moreno et al., 2005), expression of *Dll4*, *Jag1*, and *Jag2* was observed in arterial endothelial cells, but not *Dll1* expression. Furthermore, *Notch4*, which is reported to be uniformly expressed in vascular endothelial cells, was highly expressed in endothelial cells. Interestingly, *Notch1* was expressed not only in endothelial cells but also in regions reported to be hemogenic endothelium and in pre-HSCs, consistent with previous reports that Notch1 is expressed in HSC precursors (Kumano et al., 2003; Hadland et al., 2004; Souilhol et al., 2016b). Thus, in the developmental environment of HSCs—particularly in the AGM region of E10.5-E11.5 where pre-HSC-I is produced—the innermost layer, the arterial endothelium, expresses the Notch ligand. Although Notch signaling remains important, Hadland et al. recently reported on the contributions of factors other than Notch signaling in the role of the stromal environment within the *in vitro* HSC induction system (Hadland et al., 2022). This report demonstrated that Notch signaling alone could not induce HSCs and that additional signal activation from fibronectin and CXCL12 was required, supporting that multiple signaling molecules are coordinately involved in HSC development.

### BMP4

BMP4 was originally identified as being important for mesoderm development (Winnier et al., 1995; Lengerke et al., 2008) and in the promotion of EHT. BMP4 can generate hematopoietic cells from endothelial cells using an *in vitro* differentiation assay (Tsuruda et al., 2021). Furthermore, cells with activated BMP signaling in the AGM region are present in IAHCs and there is evidence that transplantation of BMP-activated cells into irradiated mice results in the engraftment of HSCs (Crisan et al., 2015).

BMP4 is abundant on the ventral side of the dorsal aorta from E10.5-E11.5 (Durand et al., 2007), while the expression of BMP inhibitory molecules is increased, and indeed, BMP signaling inhibitors have been shown to promote HSC development in an *in vitro* culture system (Souilhol et al., 2016a; McGarvey et al., 2017). Recently, we found that HSCs were induced by adding BMP4, SCF, and TPO to E9.5 hemogenic endothelium using an *in vitro* culture system (Tsuruda et al., 2024). In this culture system, it was important to remove BMP4 in the latter part of the culture. On the other hand, BMP4 was not necessary when generating HSCs from E10.5 hemogenic endothelium or pre-HSC-I (Morino-Koga et al., 2024). Therefore, it is likely that BMP4 is required for HSC development before E10.5 and gradually becomes unnecessary.

### Retinoic acid (RA)

In mouse embryos deficient in the RA synthase enzyme *Raldh2*, RA synthesis is impaired, resulting in defective hemogenic endothelium development in the yolk sac and decreased hematopoietic cellularity (Goldie et al., 2008). Raldh2-deficient mouse embryos are embryonic lethal at around E10.5 due to various morphogenetic abnormalities (Niederreither et al., 1999). The deletion of *Raldh2* specifically in *Cdh5*-expressing endothelial cells inhibits HSC production (Chanda et al., 2013). In that report, *Cdh5*-expressing cells at E10.5-E11.5 were shown to express the RA receptor, and treatment of *Raldh2*-deficient mice with an RA receptor agonist restored HSC production. Therefore, it is expected that supplementation with RA can promote HSC production *in vitro*.

### Sonic hedgehog (Shh)

The notochord is present in the dorsal periphery of the dorsal aorta. Shh supplied by the notochord is important for the differentiation of E10.5 AGM-derived cells into HSCs but not E11.5 AGM-derived cells (Souilhol et al., 2016a). Indeed, since the addition of Shh to the tissue culture systems using the E10 AGM region differentiates them into HSCs (Peeters et al., 2009), it is still unclear whether Shh is required before E9.5. Importantly, strong Shh signaling was observed in stromal cells located around the dorsal aorta (Peeters et al., 2009; Souilhol et al., 2016a). It is possible that Shh may not act directly on HSC progenitor cells but may indirectly influence HSC development by acting on surrounding cells.

### Catecholamine

The nervous system surrounding the AGM region is known to influence HSC development (Kwan et al., 2016; Lv et al., 2017). Cells of the sympathetic nervous system developing near the aorta secrete catecholamines via the transcription factor *Gata3*, which promotes HSC development in the AGM region (Fitch et al., 2012). The cell cycle regulator p57Kip2 (Cdkn1c) is also a regulator of the sympathetic nervous system; its suppression promotes sympathetic nervous system development that, in turn, promotes HSC development via catecholamines (Mascarenhas et al., 2009; Kapeni et al., 2022). Although experiments adding catecholamines have been studied in tissue culture of the E11.5 AGM region and contribute to HSC production (Fitch et al., 2012), it is unclear when catecholamines begin to affect HSC development. Further insight could be gained by additional experiments in an *in vitro* differentiation system of hemogenic endothelium or pre-HSCs.

### TPO

TPO signaling has been reported to be important for HSC maintenance and quiescence in the bone marrow and is also required for HSC engraftment capacity (Qian et al., 2007; Yoshihara et al., 2007). Yet, the source of *Tpo* is the liver, not the bone marrow (Decker et al., 2018); this suggests that TPO expression in the liver is important in maintaining HSCs. However, since HSC development in the fetal liver in *Tpo*-deficient mice is normal (Qian et al., 2007; Lee et al., 2022), evidence suggests that TPO is not required for HSC development in the fetal liver.

On the other hand, many reports have shown that TPO is important in differentiating hemogenic endothelium and pre-HSCs into HSCs in *vitro* culture (Kieusseian et al., 2012; Hadland et al., 2015; Mascarenhas et al., 2016). Recently, we reported that E11.5 pre-HSC-I can be induced into HSCs in an *in vitro* culture system without serum or feeder cells using just two signaling molecules: SCF and TPO (Morino-Koga et al., 2024). Furthermore, the cell-surface expression of MPL, the receptor for TPO, was gradually observed on E10.5 pre-HSC-I. Considering that HSC engraftment capacity was not very high, these two signaling molecules are necessary but insufficient for HSC differentiation. Interestingly, no *Tpo*-expressing cells were present in the AGM environment or surrounding tissues, and hepatoblasts in the fetal liver were the only *Tpo*-producing cells in the HSC developmental environment (Morino-Koga et al., 2024). HSCs are first produced at the AGM region but are detectable in high quantities at E12.5 in the fetal liver, suggesting that pre-HSCs can mature by migrating to the fetal liver and activating TPO signaling. As mentioned above, HSCs were produced even when TPO signaling was lost, suggesting the existence of compensatory mechanisms that activate the TPO downstream molecule, JAK2.

## Conclusion

**FIGURE 3:**
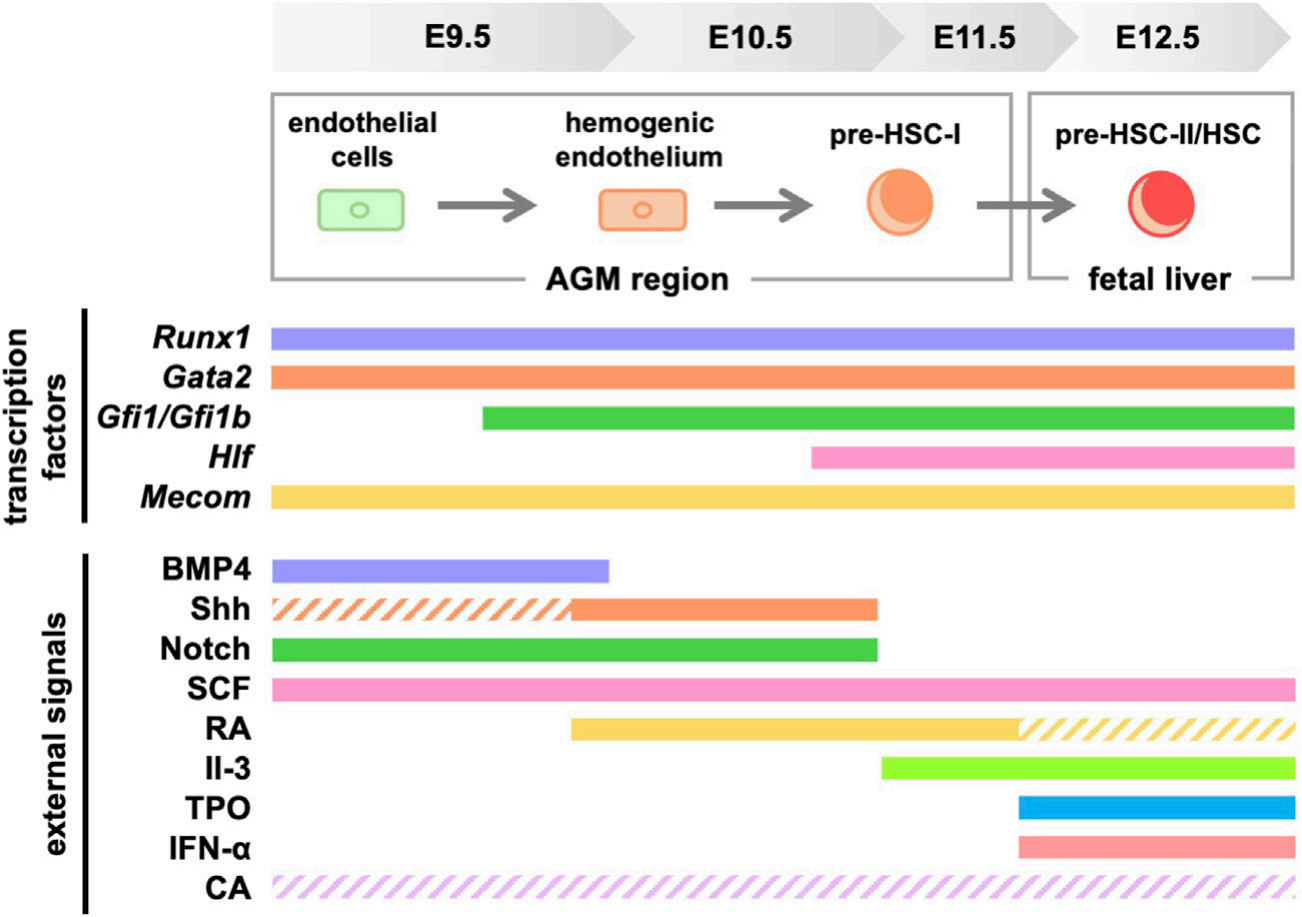
Signaling molecules and transcription factors required for HSC generation in mice. Solid lines indicate the signaling molecules involved in development, while dashed lines indicate those with unclear involvement. RA, retinoic acid; TPO, thrombopoietin; CA, catecholamine.

This review focused on signaling molecules in *vitro* differentiation systems in HSC development (Figure 3). Meanwhile, efforts are currently underway to identify transcription factors and signaling molecules that control HSC development and their engraftment in the bone marrow using human iPS cells (Doulatov et al., 2013; Piau et al., 2023; Ng et al., 2024). We believe that establishing an HSC *in vitro* differentiation system is important because it will lead to clinical applications of HSCs for hematopoietic disorders. However, in addition to the signaling molecules shown in this review, multiple environmental factors surrounding the AGM region are likely to support HSC development. For example, studies using mice and zebrafish show that HSC development is affected by blood flow-induced shear stress (Adamo et al., 2009; North et al., 2009). Therefore, it is crucial to investigate in detail which signaling molecules affect HSC development when recreating the microenvironment *in vitro*. Our serum-free culture system using only commercially available products (Morino-Koga et al., 2024) allows for the re-evaluation of key signaling molecules during HSC development under serum-containing conditions or using tissue culture systems derived from the AGM region. In the future, the identification of truly essential signaling molecules is expected to contribute to the establishment of *in vitro* differentiation systems for HSCs.

## Abbreviations

IAHC, intra-aortic hematopoietic cluster; HSC, hematopoietic stem cell; EMP, erythro-myeloid progenitor; EHT, endothelial-to-hematopoietic transition; pre-HSC, hematopoietic stem cell precursor; AGM, aorta-gonad-mesonephros.
